# Smartphone-based evaluation of static balance and mobility in long-lasting COVID-19 patients

**DOI:** 10.3389/fneur.2023.1277408

**Published:** 2023-12-11

**Authors:** Bruna Danielle Campelo Corrêa, Enzo Gabriel Rocha Santos, Anderson Belgamo, Gustavo Henrique Lima Pinto, Stanley Soares Xavier, Camilla Costa Silva, Ápio Ricardo Nazareth Dias, Alna Carolina Mendes Paranhos, André dos Santos Cabral, Bianca Callegari, Anselmo de Athayde Costa e Silva, Juarez Antônio Simões Quaresma, Luiz Fábio Magno Falcão, Givago Silva Souza

**Affiliations:** ^1^Núcleo de Teoria e Pesquisa do Comportamento, Universidade Federal do Pará, Belém, Brazil; ^2^Instituto de Ciências e Exatas e Naturais, Universidade Federal do Pará, Belém, Brazil; ^3^Instituto Federal de São Paulo, Rio Claro, Brazil; ^4^Centro de Ciências Biológicas e da Saúde, Universidade do Estado do Pará, Belém, Brazil; ^5^Núcleo de Medicina Tropical, Universidade Federal do Pará, Belém, Brazil; ^6^Instituto de Ciências da Saúde, Universidade Federal do Pará, Belém, Brazil; ^7^Instituto de Ciências da Educação, Universidade Federal do Pará, Belém, Brazil; ^8^School of Medicine, São Paulo University, São Paulo, São Paulo, Brazil; ^9^Instituto de Ciências Biológicas, Universidade Federal do Pará, Belém, Brazil

**Keywords:** long COVID-19, postural balance, mobility, smartphone, inertial sensors

## Abstract

**Background:**

SARS-CoV-2 infection can lead to a variety of persistent sequelae, collectively known as long COVID-19. Deficits in postural balance have been reported in patients several months after COVID-19 infection. The purpose of this study was to evaluate the static balance and balance of individuals with long COVID-19 using inertial sensors in smartphones.

**Methods:**

A total of 73 participants were included in this study, of which 41 had long COVID-19 and 32 served as controls. All participants in the long COVID-19 group reported physical complaints for at least 7 months after SARS-CoV-2 infection. Participants were evaluated using a built-in inertial sensor of a smartphone attached to the low back, which recorded inertial signals during a static balance and mobility task (timed up and go test). The parameters of static balance and mobility obtained from both groups were compared.

**Results:**

The groups were matched for age and BMI. Of the 41 participants in the long COVID-19 group, 22 reported balance impairment and 33 had impaired balance in the Sharpened Romberg test. Static balance assessment revealed that the long COVID-19 group had greater postural instability with both eyes open and closed than the control group. In the TUG test, the long COVID-19 group showed greater acceleration during the sit-to-stand transition compared to the control group.

**Conclusion:**

The smartphone was feasible to identify losses in the balance motor control and mobility of patients with long-lasting symptomatic COVID-19 even after several months or years. Attention to the balance impairment experienced by these patients could help prevent falls and improve their quality of life, and the use of the smartphone can expand this monitoring for a broader population.

## Introduction

Although research on coronavirus disease 2019 (COVID-19) has focused primarily on its pulmonary and cardiovascular complications, it is important to identify and study secondary manifestations of the disease (1, 2). The symptoms of SARS-CoV-2 infection extend well beyond the acute phase and may persist for many months after the patient has tested negative for COVID-19 (3–6). According to the World Health Organization, the prolongation of symptoms or the appearance of new symptoms up to 3 months after the initial infection by SARS-CoV-2, which persists for at least 2 months without any alternative explanation justifying them, characterizes the so-called post-COVID-19 condition or post-COVID-19 syndrome or still long COVID-19 (7). Although the most commonly reported symptoms are extreme fatigue, shortness of breath, and cognitive dysfunction, more than 200 different symptoms have been identified (8–10). Many of the major impairments in long COVID-19 involve the central and peripheral nervous systems (11, 12).

Long COVID-19 patients experience some level of impairment in their physical function following recovery from COVID-19 (13, 14). This impairment is evaluated using tests such as the sit-to-stand test and the six-minute walk test (13). The extensive examination of gait patterns in post-COVID patients includes various parameters such as spatial–temporal gait parameters, biomechanical factors, ground reaction forces, and changes in the gait cycle (15, 16). It is found that even those who had mild-to-moderate COVID-19 can exhibit asymmetrical gait patterns, which suggests that the impact of the disease on physical function can be long-lasting. It was observed that patients with up to 5 months since the acute phase of COVID-19 exhibited timed up and go test durations longer than those of individuals without the disease. Kinematic parameters such as walking speed and walking cadence were lower in patients with respect to the control group (13).

Among the various symptoms presented, it is noteworthy that postural control can be highly susceptible to alterations as it depends on the coordinated integration of sensory systems (visual, vestibular, and somatosensory) and the musculoskeletal system, which can present important sequelae in the long COVID-19. Although the predominant ocular manifestation during the acute phase of SARS-CoV-2 infection is conjunctivitis, it is clear that other ophthalmologic manifestations are present in the long term (17), such as loss of corneal nerve fibers, and significant changes in the pupillary response to light and impairment of pupillary microcirculation have also been observed in long COVID-19 (18–21). Some authors have shown that SARS-CoV-2 infection can spread to the middle and inner ear, causing an inflammatory response or direct effect on the epithelium, resulting in symptoms related to hearing and balance (22–25). The hyperinflammatory state induced by infection, together with lung injury, hypoxia, mitochondrial damage, renin-angiotensin system dysfunction, cerebrovascular and neuromuscular problems, peripheral neuropathies, and prolonged hospitalization, can lead to muscle damage. This can lead to fatigue, muscle weakness, myalgia, decreased physical performance, decreased functional capacity for activities of daily living, and decreased overall quality of life (26).

Previous studies have examined the effect of COVID-19 on balance control (27–30). However, the evidence available is limited to patients who have experienced postural control deficits for only a few months following infection despite all studies reporting indications of such deficits. It is currently unclear whether the observed loss of balance in individuals with long COVID-19 is permanent or if it can be reversed over longer periods. It has been 3 years since the first cases of COVID-19 were reported, but it is still uncertain whether postural control deficits persist beyond the initial few months or can be reversed over longer periods. Moreover, the current knowledge of balance control impairments is based on expensive and high-quality methods that evaluate both static and dynamic balance, which may not be available to many patients, especially those in poor and developing countries with limited resources.

Research has firmly established that smartphones can be a viable tool for evaluating balance control, offering significant advantages such as affordability and accessibility. Several studies have validated the effectiveness of smartphones in accurately assessing balance control and identifying balance deficits (31, 32). Furthermore, the use of smartphones allows for the expansion of balance evaluations to a wider population, removing barriers such as high costs and limited availability of specialized instruments.

This study aimed to compare the balance control and mobility of individuals with long-term COVID-19 (lasting several months to years) to that of healthy individuals. The comparison will be done using built-in inertial sensors on smartphones, which are cost-effective and readily available to a global population.

## Methods

### Ethical considerations

The project was approved by the Ethics and Research Committee of the Tropical Medicine Center at the Federal University of Pará (report# 61117722.3.0000.5172) and by the Ethics and Research Committee of the Biological and Health Sciences Center at the State University of Pará (approval number 61117722.3.3001.5174). Data collection was carried out between November and December 2022. All participants voluntarily agreed to participate in the research by signing the informed consent form after being fully informed about the procedures to be performed.

### Research setting and sample characterization

The clinical evaluation took place at the Outpatient Teaching Unit in Physiotherapy and Occupational Therapy (UEAFTO) of the State University of Pará (UEPA), where the COVID-19 Long Program of UEPA takes place, which serves patients with various sequelae of COVID-19. Individuals were divided into two groups. First, the long COVID-19 group, who were registered for evaluation by the aforementioned program. Patients who had not yet started the proposed treatment protocol of the program and patients who had already completed the protocol but still reported some persistent symptoms were included. Second, the control group was selected by convenience. All participants had preserved cognitive function, capacity, and conditioning to undergo the tests. Participants in the long COVID-19 group needed to have proof of a previous COVID-19 diagnosis followed by the appearance of signs or symptoms, which should have occurred at least 3 months after infection and persisted for at least 2 months. Participants could not have neurological, labyrinthine, visual, or osteomyoarticular diseases that would affect their static or dynamic balance prior to COVID-19, such as stroke, neuropathies, neurological syndromes, labyrinthine disorders, osteoarthritis, or severe or acute arthritis of the lower limbs, blindness, low vision, among others. Participants in the control group could not have had a diagnosis of COVID-19 or have presented COVID-19 without any persistent sequelae. In addition, the control group should not have any morbidity that impairs the postural stability system. All this information was collected through an interview conducted by the researchers.

### Interview and physical exam

Participants underwent an interview regarding their personal information (name, age, gender, profession, and marital status), living conditions to trace a sample profile, and the history of COVID-19 infection, hospitalization, vaccination, treatment, and sequelae. They were also asked about the presence of postural instability that emerged after the COVID-19 infection. Afterward, they underwent a basic physical examination. Muscle strength was evaluated for upper limbs (represented by shoulder flexor muscles) and lower limbs (represented by knee extensor muscles) through a manual muscle function test performed by the same evaluator, and the muscle strength was classified as “preserved” (when the strength grade was 4) and “reduced” (when the strength grade was less than 4). The range of motion (ROM) was evaluated by visual inspection of the participants, while they performed active movements of the upper limbs (shoulder flexion) and lower limbs (knee extension). When the individual could perform the complete range of motion, the ROM was classified as “preserved”; when they could not perform it, the ROM was classified as “reduced.”

Prior to the evaluation using the smartphone, in order to have an overall view of the quality of the participants’ postural stability, the traditional Romberg Test and the Sharpened Romberg Test (in Tandem position, first with the right foot behind, then with the left foot behind) were performed. Anthropometric data (height and weight) were also collected, and the body mass index (BMI) was calculated for each participant.

### Evaluation of static balance using the smartphone

For the evaluation of static balance and mobility, a Samsung mobile device, Galaxy A32 model, with Android 10 operating system was used. The mobile device had the Momentum Science app installed, which was responsible for storing readings from the inertial sensors embedded in the smartphone, which our group has used to investigate other motor functions (31, 33–35). The built-in inertial sensors in the smartphone were a triaxial accelerometer (model lsm6dsl, 16 bits, amplitude resolution ±4 g) and a triaxial gyroscope (model lsm6dsl, 16 bits, amplitude resolution 500 dps), both with a sampling rate of 50 Hz as previously done by others (36–38). The smartphone was placed on the participant’s posterior body region, at the level of the lower lumbar spine, between the L3 and L5 vertebrae, and fixed to the body using a strong elastic band attached to the waist.

For the evaluation of static balance, two recordings were performed with eyes open and two with eyes closed, each lasting 60 s. It is indicated that after 30 s recording, there is a convergence toward a stable value of the stabilometric parameters (39). Since it was found that in 60 s this convergence is higher than in 30 s, we decided to proceed with 60 s in the analysis (although it is considered reasonable also to record during 30 s). For this task, the participant was asked to stand barefoot on a flat and non-inclined surface. The participant should stand with their feet shoulder-width apart and arms relaxed at their sides with their eyes fixed on a black dot on the wall one meter away (when eyes are open). There was a 60 s interval between recordings. The total time for the static balance evaluation was 7 min.

For the evaluation of mobility, the instrumented timed up and go (iTUG) test was performed with a chair with a height between 45 and 48 cm. All participants were instructed to perform the iTUG test, in which the participant after examinator’s command should stand up without using their arms and walk for a line of 3 m, turn and walk back toward the chair. In front of the chair, the participant turns again and sit down. The participant was instructed to perform the test by walking as quickly as possible without running.

### Data analysis

**Figure 1:**
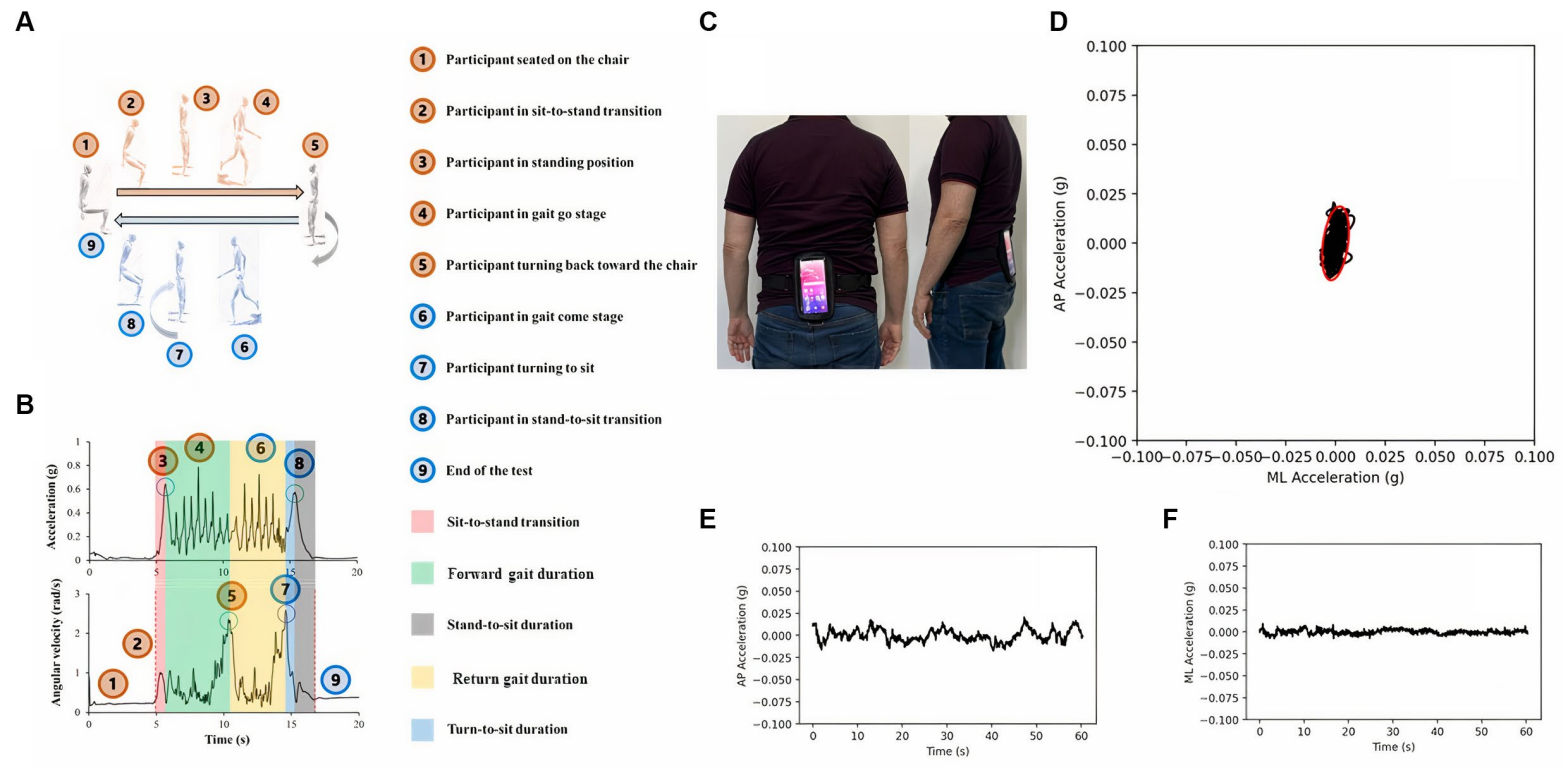
Smartphone-based mobility **(A,B)** and static balance **(C–F)** assessments. In both assessments, a smartphone was secured to a belt **(C)** and utilized to measure various aspects of physical function. For the mobility assessment, participants performed the instrumented timed up and go test, which involved rising from a chair, walking along a 3 m path, making a turn to return to the chair, walking back to the chair, and sitting down. Inertial time series data were collected during the test and subsequently analyzed to derive performance-related features. During the balance assessment, participants maintained an upright posture, while accelerometric time series data from the anteroposterior and mediolateral axes were recorded for subsequent analysis **(D–F)**.

Figure 1 displays a schematic of the balance assessment and iTUG testing procedures. For the evaluation of static balance, the accelerometric time series were extracted from the antero-posterior and medio-lateral axes to be quantified through computer routines developed in the Python computer language. The time series were processed with a zero-lag 10 Hz lowpass filter by a second-order Butterworth filter and interpolated to 100 Hz. The acceleration values were converted to gravitational units. The following parameters were calculated:

- RMS (root mean square) amplitude, in gravitational units, of the stabilogram graphs in the medio-lateral and antero-posterior axes according to Eq. 1 (40–42):

(1) $$RMS\;\mathrm{amplitude}=\sqrt{\frac{\sum_{i=1}^{n}x_{i}^{2}}{n}}$$

where 𝑥 is the value of the reading at a temporal point 𝑖 and *n* is the total number of temporal points in the measurement.

- Path, in gravitational units, according to Eq. 2 (42):

(2) $$\mathrm{Path}=\overset{n}{\underset{i=1}{\sum }}\sqrt{AP_{i}^{2}+ML_{i}^{2}}$$

where 𝐴𝑃 and 𝑀𝐿 are the values of the readings at the temporal point 𝑖 in the antero-posterior and medio-lateral displacement, respectively, and 𝑁 is the total number of temporal points in the measurement.

- 95% confidence ellipse area, in square gravitational units. The confidence ellipse area is defined as the area of the ellipse that contains the true mean AP and ML with a probability of 95% (43). For that, we calculated the covariance matrix of the data and found the eigenvalues and eigenvectors of that matrix. The major and minor axes of the ellipse correspond to the directions associated with the largest and smallest eigenvalues, and the ellipse captures the uncertainty or confidence in the data’s distribution in those directions.
- Median frequency, in Hz, which was calculated by applying a Fourier transform on the accelerometric time series of the antero-posterior and medio-lateral axes and represents the frequency at which 50% of the total power in the spectrum is contained below this frequency, and 50% is contained above it (41).

The algorithm to extract the features of the balance evaluation is shown in the Supplementary material.

For the evaluation of mobility, accelerometric and gyroscopic time series of the three analyzed axes were extracted. For both sensors, the norm was calculated using Eq. 3 (44):

(3) $$\mathrm{norm}=\sqrt{x^{2}+y^{2}+z^{2}}$$

where *x*, *y*, and *z* are the inertial time series recorded in the three axes of analysis.

We developed an algorithm written in Python language based on previous studies (13, 44) to find transient components of the inertial waveforms. In the present approach, we focused on analyzing the norm vector of each inertial sensor. After visual evaluation of the parameters found by the algorithm. The algorithm and the rationale for it are available in the Supplementary material. It was possible to extract quantitative inertial parameters during the iTUG test: total duration, sit-to-stand transition duration, forward gait duration, return gait duration, stand-to-sit duration, acceleration peak to standing, acceleration peak to sitting, angular velocity peak in the return turn, angular velocity peak in the pre-sitting turn, standing jerk, and sitting jerk (13, 44–46). Supplementary material shows the criteria to calculate each parameter.

### Statistical analysis

Data analysis was processed using the software Jamovi 2.2.5.0. A significance level of 5% (*p*-value<0.05) was considered for all analyses. The *p*-value was corrected for false discovery rate using the Benjamini–Hochberg procedure. To test the statistical difference between the data from both groups (age, BMI, static balance with eyes open, with eyes closed, and dynamic balance), the data distribution was first evaluated using the Shapiro–Wilk test. Then, an unpaired *t*-test was used with Welch correction when the data had a normal distribution, and the Mann–Whitney test was used when the normality assumptions were violated by the Shapiro–Wilk test. To compare the proportion of males and females between the COVID-19 group and the control group, the Yates-corrected chi-square test was used.

## Results

Table 1 shows demographic data from both groups, and it was observed that they were matched for age, BMI, and proportion of men and women.

**Table 1:** Demographic data from both groups. Values representing median (interquartile range).

Variables	Control group	Long COVID-19 group	*p*-value
*Sex*			
Male/Female	10/31	11/21	0.5
Age (years)	50 (9)	50.5 (10.5)	0.93
BMI (kg/m^2^)	26.4 (6.8)	25.7 (3.2)	0.75

Values represent median (interquartile range). BMI, body mass index.

Table 2 provides information on the natural history of COVID-19 for participants in the long COVID group and the control group (as some participants in the latter group presented with COVID-19). It can be observed that the majority of patients in the long COVID group were not hospitalized during the acute phase of COVID-19 and all of them received at least one dose of COVID-19 vaccination. As for the control group, 25 people had COVID-19 and only one did not receive any dose of COVID-19 vaccine.

**Table 2:** Natural history related to COVID-19 from both groups.

COVID-19 history	Control group (*n* = 32)	Long COVID-19 group (*n* = 41)
*Long COVID-19 duration (months)*	–	30 (8)
*Positivity to COVID-19*	25	41
*Hospital stays*	0	5
*Vaccination*		
0 dose	1	0
1 dose	1	2
2 doses	8	1
3 doses	8	20
4 doses	14	18

Long COVID-19 participants were also questioned about the symptoms presented since the infection. Sequelae of various types were recorded, and all participants presented with more than one symptom simultaneously. The symptoms were numerous and were divided into categories (types of symptoms), as presented in Table 3.

**Table 3:** Symptoms observed in the long Covid-19 group (*n* = 41).

Symptoms	*N*
Pneumological complications	19
Cardiovascular complications	8
Neurological complications	32
Musculoskeletal complications	32
Oto-labyrinthine complications	23
Psychiatric complications	23
Gastrointestinal complications	3
Metabolic complications	3
Dermatological complications	4
Generalized fatigue	31
Loss of balance	22
Hyposmia/ageusia	16

*n*, number of participants.

Regarding the physical exam, a significant difference in muscular strength was observed between the long COVID group and the control group. In the traditional and sensitized Romberg tests (both Tandem on the right and on the left), a large difference was also noted. Many of the long COVID-19 patients presented with Romberg sign positive. However, there was no significant difference between the groups in the evaluation of range of motion (Table 4).

**Table 4:** Number of participants presenting the different features in the physical exam.

	Number of participants		
Physical exam	Control group	Long COVID-19 group	*p*-value
Preserved upper limb muscular strength	32	13	<0.001*
Preserved lower limb muscular strength	32	17	<0.0001*
Preserved range of motion of upper limbs	32	38	0.25
Preserved range of motion of lower limbs	32	41	0.99
Positive Romberg test	0	11	0.002*
Positive Sharpened Romberg test	1	33	<0.001*

*p-adjusted < 0.05.

In comparing the measured data in the assessment of static balance with eyes open and eyes closed between groups, there was a significant statistical difference between the groups in the parameters obtained in the time domain, both in the experimental condition with eyes open and with eyes closed. In general, the long COVID group presented with greater amplitudes of body oscillations than the control group in both conditions (Table 5).

**Table 5:** Comparison of static balance features in open and closed eyes condition.

Static balance	Control group	Long COVID-19 group	*p*-value
*Open eyes condition*			
Path (g)	23.76 (6.6)	27.66 (5.9)	0.025*
RMS ML (×10^−3^) (g)	1.65 (0.5)	1.99 (0.8)	0.006*
RMS AP (×10^−3^) (g)	3.04 (0.8)	5.37 (1.3)	<0.001*
Area (×10^−3^) (g^2^)	16.77 (6.1)	21.36 (9.1)	0.025*
Median frequency ML (Hz)	4.49 (0.8)	4.33 (1.4)	0.056
Median frequency AP (Hz)	1.86 (1)	1.57 (0.9)	0.059
*Closed eyes condition*			
Path (g)	26.51 (8.9)	32.58 (16.2)	0.009*
RMS ML (×10^−3^) (g)	1.76 (0.6)	2.23 (1.4)	0.014*
RMS AP (×10^−3^) (g)	5.07 (1.8)	6.26 (3.1)	0.02*
Area (×10^−3^) (g^2^)	19.25 (6.6)	22.99 (16.4)	0.041
Median frequency ML (Hz)	4.41 (0.9)	4.16 (1.5)	0.41
Median frequency AP (Hz)	1.69 (0.6)	1.4 (0.9)	0.32

Values represent median (interquartile range). g, gravity; AP, anteroposterior; ML, mediolateral; F50, median frequency; *p-adjusted < 0.05.

The comparison of parameters related to the evaluation during the dynamic balance task showed that there was only a statistical difference in the acceleration peak during the sit-to-stand variation in the iTUG test. All other parameters did not present a statistical difference (Table 6).

**Table 6:** Comparison of dynamic balance features in open and closed eyes condition.

Dynamic balance	Control group	Long COVID-19 group	*p*-value
Total duration (s)	13.2 (2.6)	13.6 (3.8)	0.36
Sit-to-stand duration (s)	1.0 (0.2)	1.0 (0.35)	0.12
Forward gait duration (s)	4.9 (0.97)	4.9 (1.5)	0.92
Return gait duration (s)	4.4 (1.5)	4.6 (2)	0.82
Stand-to-sit duration (s)	1.57 (0.42)	1.45 (0.54)	0.83
Acceleration peak to stand (g)	0.43 (0.09)	0.51 (0.11)	0.009*
Acceleration peak to sit (g)	0.49 (0.08)	0.52 (0.11)	0.28
Angular velocity peak during return turn (rad/s)	2.4 (0.49)	2.4 (0.72)	0.47
Angular velocity peak during pre-sitting turn (rad/s)	3.0 (0.72)	2.8 (1.1)	0.32
Standing jerk (g/s)	0.43 (0.11)	0.46 (0.13)	0.28
Sitting jerk (g/s)	0.33 (0.1)	0.36 (0.1)	0.37

Values represent median (interquartile range). rad, radians; s, second; g, gravity; *p-adjusted < 0.05.

## Discussion

Using inertial sensors in smartphones, the study found that COVID-19 patients with symptoms even years after the initial COVID-19 infection had significant balance impairments compared to a control group. Additionally, the static balance of these patients was more compromised than their mobility. It is worth highlighting that the balance deficits persisted for a longer duration than the literature has previously reported.

Postural balance relies on the integration of sensory and motor systems (47). These systems are commonly affected in the post-COVID-19 state (4, 21, 24). The balance losses observed in the participants of this study may have multifactorial explanations. Participants showed symptoms and signs of functional losses from different systems involved in postural control, such as the vestibular system, somatosensory system, and muscular system. Our study found a high incidence of joint stiffness, arthralgia, and myalgia in the long COVID-19 group. Additionally, the muscle strength of the affected group was significantly lower than that of the healthy control group. Moreover, fatigue in most of the affected group may impede the maintenance of a standing position.

Prior research has noted impairments in both static and dynamic balance among COVID-19 patients (27–30). Computerized dynamic posturography was used to assess thirty-five patients who had recovered from COVID-19 (between 22 and 124 days after the disease onset) using the sensory organization test protocol to differentiate the visual, vestibular, and somatosensory components of balance control (28). The COVID-19 group demonstrated a significant loss in dynamic balance compared to the control group, with the greatest difference noted in the visual component of the test. The dynamic balance control of thirty-three patients within 2 to 4 weeks of SARS-CoV-2 infection was evaluated through stabilography which employs the rambling-trembling method (29). No statistically significant difference was found between healthy subjects and individuals in the post-COVID-19 state. Patients who presented olfactory changes tended to have lower postural control than those without such alteration. Long COVID-19 patients, with at least 12 weeks after SARS-CoV-2 infection, were found to have a strong correlation between muscle weakness (measured by dynamometry), generalized fatigue (measured by the Functional Assessment of Chronic Illness Therapy Fatigue questionnaire), and balance deficit (measured by the Berg and Tinetti scales) (30). After approximately 4.5 months of primary infection, long COVID-19 patients were observed to have static balance losses using a force platform with bipodal and unipodal support, and worse performance in dynamic tasks such as the timed up and go test, 6 min walk test, sit-to-stand test, and 15 s step test (27). Using multiple inertial sensors, it was observed that patients, 8–25 weeks after hospital discharge, experienced changes in both their range of motion and the time taken to complete the TUG task (13). Our results did not show those differences in the duration of the iTUG phases and in total duration. This difference between the two studies may be due to some degree of mobility recovery of the subjects of our study as they had a longer median duration (30 months vs. 8–5 weeks). Our study revealed that individuals with long COVID-19 exhibited higher acceleration during the transition from a standing to a sitting position compared to the control group. This is interpreted as an indicator of impaired control during the descent onto the chair, where the patient tends to release and fall into the seat. In contrast, a healthy individual typically executes the descent onto the chair with deceleration, facilitated by the eccentric contraction of the gluteus maximus and quadriceps muscles. Muscle tissue injury in COVID-19 is probably explained by a multifactorial mechanism (4).

While we have observed that the Romberg test has already shown significant differences and is relatively easy to administer, it is important to note that its results are binary, classifying as either “normal” or “altered” and do not provide differentiation among different levels of disturbances. In contrast, the sensor data yields quantitative outcomes, enabling us to classify and differentiate various levels of disturbance severity.

Comparing our data to the literature to check the consistency, we observed that for the controls, the stabilometric values as well as duration of the different stages of iTUG are similar to previously reported values for healthy adults (48–51) as well as for COVID-19 patients (13).

This study varies from others that explored balance control in long COVID-19 patients in some aspects. To begin with, the long COVID-19 patients in this study have a longer duration since their initial SARS-CoV-2 infection in comparison to similar studies. The study’s samples have a median duration of 30 months since the infection onset and have been vaccinated with different doses, hence being more relevant to the current conditions. Thus, this initial variation provides an indication that the balance losses that long COVID-19 patients experience might have a longer duration or even be permanent. Another divergence is the use of inertial sensors, integrated into smartphones, to assess the static and dynamic balance control parameters. This approach was able to reveal comparable outcomes with the previous studies that adopted gold standard methods for evaluating balance control. The clinical reliability of the smartphones’ embedded inertial sensors in identifying balance losses in long COVID-19 patients could facilitate more extensive reach of such a diagnosis since it is low-cost and easily transported, possibly increasing the monitoring of balance loss coverage, in particular among low-income populations.

The number of COVID-19 vaccine doses received by the participants varied within the sample, which may be viewed as a limitation of this research. Nevertheless, a large number of participants received at least two of the four doses distributed by the Brazilian Ministry of Health. Furthermore, the absence of control over the medication used by long COVID-19 patients after the onset of the disease is a limitation. Another limitation of the present study is that the instrumented TUG algorithm, even if it was based on previous studies, has not been validated yet with measures obtained from gold-standard methods, such as video capture.

Long COVID-19 is a recent and still not well-understood condition. Numerous studies are currently investigating the condition to determine its causes, risk factors, and potential treatments (52). This study has demonstrated that patients can experience long-term functional consequences following initial infection with COVID-19. These results may assist in the development of public policies aimed at monitoring balance and fall prevention in these patients.

## Data availability statement

The raw data supporting the conclusions of this article will be made available by the authors, without undue reservation.

## Ethics statement

The studies involving humans were approved by Ethics and Research Committee of the Tropical Medicine Center at the Federal University of Pará (report# 61117722.3.0000.5172) and by the Ethics and Research Committee of the Biological and Health Sciences Center at the State University of Pará (approval number 61117722.3.3001.5174). The studies were conducted in accordance with the local legislation and institutional requirements. The participants provided their written informed consent to participate in this study.

## Author contributions

BC: Conceptualization, Formal analysis, Investigation, Methodology, Writing – original draft, Writing – review & editing. ES: Formal analysis, Software, Writing – review & editing. AB: Software, Writing – review & editing. GP: Software, Writing – review & editing. SX: Investigation, Writing – review & editing. CS: Investigation, Writing – review & editing. ÁD: Data curation, Investigation, Writing – review & editing. AP: Investigation, Writing – review & editing. ACa: Investigation, Software, Writing – review & editing. BC: Funding acquisition, Methodology, Software, Writing – review & editing. ACo: Funding acquisition, Software, Supervision, Writing – review & editing. JQ: Conceptualization, Data curation, Funding acquisition, Investigation, Writing – original draft, Writing – review & editing. LF: Conceptualization, Data curation, Funding acquisition, Investigation, Writing – original draft, Writing – review & editing. GS: Conceptualization, Data curation, Formal analysis, Funding acquisition, Methodology, Project administration, Resources, Software, Supervision, Visualization, Writing – original draft, Writing – review & editing.
