# Supplementary material for: Smartphone-based evaluation of static balance and mobility in long-lasting COVID-19 patients
Source: Front Neurol. 2023 Dec 11;14:1277408. doi: 10.3389/fneur.2023.1277408 (PMC10750373; doi:10.3389/fneur.2023.1277408)
Supplement: Supplementary file 1 [file Data_Sheet_1.PDF]

SUPPLEMENTARY MATERIAL 1:

Algorithm for assessing static balance control and the instrumented Timed Up and Go using the smartphone application Momentum Science, implemented in the Python language. Before running the code, it is necessary to install Streamlit, Pandas, NumPy, Matplotlib, and SciPy packages. It is preferable to run this code in Visual Studio Code.

The code generates a web application. The user should write in the terminal the command: **streamlit run** *python filename* (extension .py)

```
# import the packages
import streamlit as st
import pandas as pd
import numpy as np
import matplotlib.pyplot as plt
from scipy import signal
from scipy.signal import butter, filtfilt
import scipy.interpolate
from scipy.signal import find_peaks
from scipy.spatial import ConvexHull

#fast fourier transform for spectral analysis
def balance_fft(data):
    fs = 100  # Sampling frequency
    # Perform FFT
    fft_result = np.fft.fft(data)
    N = len(fft_result)
    frequencies = np.fft.fftfreq(N, 1/fs)
    c = 0
    a = 0
    spectrum_amplitude = []
    freq = []
    for i in frequencies:
        c = c + 1
        if i >= 0:
            spectrum_amplitude.append(np.abs(fft_result)[c])
            freq.append(i)
            a = a +
    a = 0
    spectrum_amplitude[0] = 0
    for i in freq:
        a = a + 1
        if i > 0.5:
            f1 = a
            break
    a = 0
    for i in freq:
        a = a + 1
        if i > 2:
            f2 = a
```

```
            break
    a = 0
    for i in freq:
        a = a + 1
        if i > 6:
            f3 = a
            break
    total_spectal_energy = sum(spectrum_amplitude[0:f3])
    energy = 0
    c = 1
    while energy < total_spectal_energy/2:
        energy = np.sum(spectrum_amplitude[0:c])
        c = c + 1
    median_frequency = freq[c]
    LF_energy = sum(np.abs(spectrum_amplitude[0:f1]))
    MF_energy = sum(np.abs(spectrum_amplitude[f1:f2]))
    HF_energy = sum(np.abs(spectrum_amplitude[f2:f3]))

    return freq, spectrum_amplitude, median_frequency, LF_energy,
MF_energy, HF_energy

# function for butterworth filter
def butterworth_filter(data, cutoff, fs, order=4, btype='low'):
    nyquist = 0.5 * fs
    normal_cutoff = cutoff / nyquist
    b, a = butter(order, normal_cutoff, btype, analog=False)
    y = filtfilt(b, a, data)
    return y

# function to fit the ellipse in the statokinesiogram plot
def set_ellipse(fpML, fpAP):
    points = np.column_stack((fpML, fpAP))
    hull = ConvexHull(points)

    # Get the boundary points of the convex hull
    boundary_points = points[hull.vertices]

    # Calculate the centroid of the boundary points
    centroidx = np.mean(fpML)
    centroidy = np.mean(fpAP)
    centroid = centroidx, centroidy

    # Calculate the covariance matrix of the boundary points
    covariance = np.cov(boundary_points, rowvar=False)

    # Calculate the eigenvalues and eigenvectors of the covariance matrix
    eigenvalues, eigenvectors = np.linalg.eig(covariance)

    # Calculate the major and minor axis of the ellipse
```

```
    major_axis = np.sqrt(eigenvalues[0]) * np.sqrt(-2 * np.log(1 -
0.95))/2
    minor_axis = np.sqrt(eigenvalues[1]) * np.sqrt(-2 * np.log(1 -
0.95))/2

    # Finding the ellipse coordinates
    angle = np.degrees(np.arctan2(*eigenvectors[:, 0][::-1]))
    area = np.pi*major_axis*minor_axis
    num_points = 101  # 360/100 + 1
    ellipse_points = np.zeros((num_points, 2))
    a = 0
    for i in np.arange(0, 361, 360 / 100):
        ellipse_points[a, 0] = centroid[0] + major_axis *
np.cos(np.radians(i))
        ellipse_points[a, 1] = centroid[1] + minor_axis *
np.sin(np.radians(i))
        a += 1
    angle_deg = -angle
    angle_rad = np.radians(angle_deg)

    # Rotating the ellipse
    R = np.array([[np.cos(angle_rad), -np.sin(angle_rad)],
                  [np.sin(angle_rad), np.cos(angle_rad)]])
    ellipse_points = np.dot(ellipse_points, R)
    return ellipse_points, area, angle_deg, major_axis, minor_axis


# Set the page expanded configuration with two tabs
st.set_page_config(layout="wide", initial_sidebar_state="expanded")
tab1, tab2 = st.tabs(["Balance", "iTUG"])

# Algorithm to proceed static balance control analysis
with tab1:
    st.title("Smartphone-based balance test")
    t1, t2, t3 = st.columns([1, 1, 1])

    # Acceleration file upload button
    uploaded_acc = st.file_uploader("Upload accelerometer text file",
type=["txt"],)

    # Check if a file has been uploaded
    if uploaded_acc is not None:

        # Read and display the data from the uploaded CSV file
        if uploaded_acc is not None:
            custom_separator = ';'

            # Allocation of the data to the variables
            df = pd.read_csv(uploaded_acc, sep=custom_separator)
```

```
            t = df.iloc[:, 0]
            x = df.iloc[:, 1]
            y = df.iloc[:, 2]
            z = df.iloc[:, 3]
            time = t
            AP = z

            # Selection of ML axis between x and y axis. The one that
recorded the gravity acceleration is excluded and the other is the ML
axis
            if np.mean(x) > np.mean(y):
                ML = y
            else:
                ML = x

            # Pre-processing data: AP and ML channels were detrended and
normalized to gravity acceleration
            if np.max(x) > 9 or np.max(y) > 9:
                AP = signal.detrend(AP/9.81)
                ML = signal.detrend(ML/9.81)
            else:
                AP = signal.detrend(AP)
                ML = signal.detrend(ML)

            # Pre-processing data: interpolating to 100 Hz
            interpf = scipy.interpolate.interp1d(time, AP)
            time_ = np.arange(start=time[0], stop=time[len(time)-1],
step=10)
            AP_ = interpf(time_)
            xAP, yAP = time_/1000, AP_
            yAP = butterworth_filter(yAP, 10, 100, order=2, btype='low')
            interpf = scipy.interpolate.interp1d(time, ML)
            time_ = np.arange(start=time[0], stop=time[len(time)-1],
step=10)
            ML_ = interpf(time_)
            xML, yML = time_/1000, ML_
            yML = butterworth_filter(yML, 6, 100, order=4, btype='low')

            # norm calculation
            norm = np.sqrt(yAP**2+yML**2)
            length_balance = len(yML)

            # Creating controls to interacts with the plots
            with t3:
                slider_min = st.number_input(
                    "Select the recording ONSET", min_value=1,
max_value=length_balance-1, step=1, value=1)
                slider_max = st.number_input(
```

```
                    "Select the recording OFFSET", min_value=1,
max_value=length_balance-1, value=length_balance-1, step=1)
                initial_state = True
                checkbox_1 = st.checkbox(
                    "Show whole recording", value=initial_state)
                checkbox_2 = st.checkbox(
                    "Show analysed period", value=initial_state)

            # Ellipse fitting and features extraction from ellipse
            ellipse_fit, area_value, angle_deg_value, major_axis_value,
minor_axis_value = set_ellipse(
                yML[slider_min:slider_max], yAP[slider_min:slider_max])

            # Extracting features: total deviation, rmsAP, rmsML
            total_deviation = sum(np.sqrt(norm[slider_min:slider_max]))
            rmsAP =
np.sqrt(np.mean(np.square(yAP[slider_min:slider_max])))
            rmsML =
np.sqrt(np.mean(np.square(yML[slider_min:slider_max])))
            frequencies, spectrum_amplitude_ML, median_frequency_ML,
LF_energy_ML, MF_energy_ML, HF_energy_ML = balance_fft(
                yML[slider_min:slider_max])
            frequencies, spectrum_amplitude_AP, median_frequency_AP,
LF_energy_AP, MF_energy_AP, HF_energy_AP = balance_fft(
                yAP[slider_min:slider_max])

            # Plotting statokinesiogram
            with t1:
                plt.figure(figsize=(5, 5))
                if checkbox_1 == True:
                    plt.plot(yML, yAP, 'grey')
                if checkbox_2 == True:
                    plt.plot(yML[slider_min:slider_max],
                             yAP[slider_min:slider_max], 'k')
                plt.plot(ellipse_fit[:, 0], ellipse_fit[:, 1], 'r')
                plt.fill(ellipse_fit[:, 0], ellipse_fit[:,
                         1], color='tomato', alpha=0.5)
                plt.xlabel('ML Acceleration (g)')
                plt.ylabel('AP Acceleration (g)')
                plt.ylim(-0.1, 0.1)
                plt.xlim(-0.1, 0.1)
                st.pyplot(plt)
                plt.figure(figsize=(5, 5))
                plt.plot(frequencies, spectrum_amplitude_AP, 'k')
                plt.xlabel('Temporal frequency (Hz)')
                plt.ylabel('AP Acceleration power (g^2)')
                plt.xlim(0, 6)
                st.pyplot(plt)
```

```
# Stabilogram plots
with t2:
    plt.figure(figsize=(5, 1.75))
    if checkbox_1 == True:
        plt.plot(xAP, yAP, 'grey')
    if checkbox_2 == True:
        plt.plot(xAP[slider_min:slider_max],
                 yAP[slider_min:slider_max], 'k')
    plt.xlabel('Time (s)')
    plt.ylabel('AP Acceleration (g)')
    plt.ylim(-0.1, 0.1)
    st.pyplot(plt)
    plt.figure(figsize=(5, 1.75))
    if checkbox_1 == True:
        plt.plot(xML, yML, 'grey')
    if checkbox_2 == True:
        plt.plot(xML[slider_min:slider_max],
                 yML[slider_min:slider_max], 'k')
    plt.xlabel('Time (s)')
    plt.ylabel('ML Acceleration (g)')
    plt.ylim(-0.1, 0.1)
    st.pyplot(plt)
    plt.figure(figsize=(5, 5))
    plt.plot(frequencies, spectrum_amplitude_ML, 'k')
    plt.xlabel('Temporal frequency (Hz)')
    plt.ylabel('ML Acceleration power (g^2)')
    plt.xlim(0, 6)
    st.pyplot(plt)

# Printing of the features values
with t3:
    st.markdown("**Outcomes**")
    st.text('RMS AP (g) = ' + str(round(rmsAP, 5)))
    st.text('RMS ML (g) = ' + str(round(rmsML, 5)))
    st.text('Total deviation (g) = ' +
            str(round(total_deviation, 3)))
    st.text('Area (g^2) = ' + str(round(area_value, 5)))
    st.text('Major axis (g) = ' + str(round(major_axis_value,
5)))
    st.text('Minor axis (g) = ' + str(round(minor_axis_value,
5)))
    st.text('Rotation angle (deg) = ' +
            str(round(angle_deg_value, 2)))
    st.text('Median frequency AP (Hz) = ' +
            str(round(median_frequency_AP, 2)))
    st.text('Median frequency ML (Hz) = ' +
            str(round(median_frequency_ML, 2)))
    st.text('Power low frequency AP (g^2) = ' +
            str(round(LF_energy_AP, 2)))
```

```
                st.text('Power medium frequency AP (g^2) = ' +
                        str(round(MF_energy_AP, 2)))
                st.text('Power high frequency AP (g^2) = ' +
                        str(round(HF_energy_AP, 2)))
                st.text('Power low frequency ML (g^2) = ' +
                        str(round(LF_energy_ML, 2)))
                st.text('Power medium frequency ML (g^2) = ' +
                        str(round(MF_energy_ML, 2)))
                st.text('Power high frequency ML (g^2) = ' +
                        str(round(HF_energy_ML, 2)))

# Algorithm to proceed iTUG analysis. All the analysis we've done in the iTUG was based on finding six transient events related to different stages of the task. (i) Task onset; (ii) Turn to return; (iii) Turn to sit; (iv) Sit-to-stand transition; (v) Stand-to-sit transition; and (vi) Task offset
with tab2:
    st.title("Smartphone-based Timed Up and Go Test")
    t1, t2, t3 = st.columns([1, 1.75, 1])

    # Create acceleration and gyroscope file upload buttons
    uploaded_acc_iTUG = st.file_uploader(
        "Upload accelerometer iTUG text file", type=["txt"],)
    uploaded_gyro_iTUG = st.file_uploader(
        "Upload gyroscope iTUG text file", type=["txt"],)

    if uploaded_acc_iTUG is not None:
        # Allocation of the acceleration data to the variables
        if uploaded_acc_iTUG is not None:
            custom_separator = ';'
            df = pd.read_csv(uploaded_acc_iTUG, sep=custom_separator)
            t = df.iloc[:, 0]
            x = df.iloc[:, 1]
            y = df.iloc[:, 2]
            z = df.iloc[:, 3]
            time = t

            # Pre-processing data: All channels were detrended, normalized to gravity acceleration, and interpolated to 100 Hz
            if np.max(x) > 9 or np.max(y) > 9 or np.max(z) > 9:
                x = signal.detrend(x/9.81)
                y = signal.detrend(y/9.81)
                z = signal.detrend(z/9.81)
            else:
                x = signal.detrend(x)
                y = signal.detrend(y)
                z = signal.detrend(z)
            interpf = scipy.interpolate.interp1d(time, x)
```

```
            time_ = np.arange(start=time[0], stop=time[len(time)-1],
step=10)
            x_ = interpf(time_)
            t, x = time_/1000, x_
            interpf = scipy.interpolate.interp1d(time, y)
            time_ = np.arange(start=time[0], stop=time[len(time)-1],
step=10)
            y_ = interpf(time_)
            t, y = time_/1000, y_
            interpf = scipy.interpolate.interp1d(time, z)
            time_ = np.arange(start=time[0], stop=time[len(time)-1],
step=10)
            z_ = interpf(time_)
            t, z = time_/1000, z_

            # Calculating acceleration data norm (Ko et al., 2022)
            norm_waveform = np.sqrt(x**2+y**2+z**2)

            # Filtering acceleration data norm
            norm_waveform = butterworth_filter(
                norm_waveform, 10, 100, order=2, btype='low')

        # Allocation of the gyroscope data to the variables
        if uploaded_gyro_iTUG is not None:
            custom_separator = ';'
            df_gyro = pd.read_csv(uploaded_gyro_iTUG,
sep=custom_separator)
            t_gyro = df_gyro.iloc[:, 0]
            x_gyro = df_gyro.iloc[:, 1]
            y_gyro = df_gyro.iloc[:, 2]
            z_gyro = df_gyro.iloc[:, 3]
            time_gyro = t_gyro

            # Pre-processing data: All channels were detrended, and
interpolated to 100 Hz
            x_gyro = signal.detrend(x_gyro)
            y_gyro = signal.detrend(y_gyro)
            z_gyro = signal.detrend(z_gyro)
            interpf = scipy.interpolate.interp1d(time_gyro, x_gyro)
            time_gyro_ = np.arange(
                start=time_gyro[0], stop=time_gyro[len(time_gyro)-1],
step=10)
            x_gyro_ = interpf(time_gyro_)
            t_gyro, x_gyro = time_gyro_/1000, x_gyro_
            interpf = scipy.interpolate.interp1d(time_gyro, y_gyro)
            time_gyro_ = np.arange(
                start=time_gyro[0], stop=time_gyro[len(time_gyro)-1],
step=10)
            y_gyro_ = interpf(time_gyro_)
```

```
            t_gyro, y_gyro = time_gyro_/1000, y_gyro_
            interpf = scipy.interpolate.interp1d(time_gyro, z_gyro)
            time_gyro_ = np.arange(
                start=time_gyro[0], stop=time_gyro[len(time_gyro)-1],
step=10)
            z_gyro_ = interpf(time_gyro_)
            t_gyro, z_gyro = time_gyro_/1000, z_gyro_

            # Calculating norm for angular velocity
            norm_waveform_gyro = np.sqrt(x_gyro**2+y_gyro**2+z_gyro**2)

            # Filtering norm for acceleration
            norm_waveform_gyro = butterworth_filter(
                norm_waveform_gyro, 10, 100, order=2, btype='low')

            # Creating controls to interacts with the plots
            with t1:
                # Create slider widgets to set limits of baselines for
the test onset and test offset
                st.markdown("**Set baseline intervals**")
                length = len(norm_waveform_gyro)
                slider_baseline1 = st.number_input(
                    "Select the baseline_min ONSET", min_value=1,
max_value=length, step=1, value=50)
                slider_baseline2 = st.number_input(
                    "Select the baseline_max ONSET", min_value=1,
max_value=length, value=100, step=1)
                slider_baseline3 = st.number_input(
                    "Select the baseline_min OFFSET", min_value=1,
max_value=length, value=length-150, step=1)
                slider_baseline4 = st.number_input(
                    "Select the baseline_max OFFSET", min_value=1,
max_value=length, value=length-100, step=1)

                # We used the data from gyroscope to find the onset of
the sitting to standing transition following Van Lummel et al. (2013)
recommendation. We calculated the first derivative of the norm because we
observed that it has less variability than the norm and it would
facilitate to find the moment of the deflection from sitting to standing
position. The basic idea to find the onset of the task was to choose a
period during the pre-standing as baseline. Then, we calculated the
average and standard deviation of the baseline from gyroscope first
derivative vector and searched for the moment that the vector value
exceeded the mean plus 4*standard deviation
            firstDerivative = np.diff(norm_waveform_gyro)

            # Setting the limits of the baseline to find the task onset.
It is selected a range in the beginning of the recording
            if slider_baseline1 < slider_baseline2:
```

```
                avg_firstderivative = np.mean(
                    firstDerivative[slider_baseline1:slider_baseline2])
                std_firstderivative = np.std(
                    firstDerivative[slider_baseline1:slider_baseline2])
                loc_onset = slider_baseline2

                # Finding the task onset
                for i in firstDerivative[slider_baseline2:length-
slider_baseline2]:
                    if i < avg_firstderivative + 4 * std_firstderivative:
                        loc_onset = loc_onset + 1
                    else:
                        break

            # Setting the limits of the baseline to find the task onset.
It is selected a range in the end of the recording.
            if slider_baseline3 < slider_baseline4:
                avg_firstderivative_offset = np.mean(
                    firstDerivative[slider_baseline3:slider_baseline4])
                std_firstderivative_offset = np.std(
                    firstDerivative[slider_baseline3:slider_baseline4])
                loc_offset = slider_baseline3

                # Finding the task offset
                for i in reversed(firstDerivative[1:slider_baseline3]):
                    if i < avg_firstderivative_offset +
4*std_firstderivative_offset:
                        loc_offset = loc_offset - 1
                    else:
                        break

                # Setting the sliders with onset and offset positions
                st.markdown("**Manual adjustments**")
                slider_onset = st.number_input(
                    "Select the onset", min_value=1, max_value=length,
value=loc_onset, step=1)
                slider_offset = st.number_input(
                    "Select the offset", min_value=1, max_value=length-1,
value=loc_offset, step=1)

                # Next step is to find the angular velocity peak during
the return turn and pre-sitting turn. For that, we found the amplitude
peaks and the position of the two largest amplitudes in the gyroscope
norm. To indicate which component is each amplitude we compared the
location in the vector. The earlier is from the return turn and the later
is from the pre-sitting turn
                peaks, _ = find_peaks(norm_waveform_gyro, height=0.5)
                amplitude = norm_waveform_gyro[peaks]
                amplitude = sorted(amplitude, reverse=True)
```

```
                a = 0
                for i in norm_waveform_gyro:
                    a = a + 1
                    if i == amplitude[0]:
                        loc1 = a
                        latency1 = t_gyro[a]
                        amplitude1 = norm_waveform_gyro[a]
                        break
                a = 0
                for i in norm_waveform_gyro:
                    a = a + 1
                    if i == amplitude[1]:
                        loc2 = a
                        latency2 = t_gyro[a]
                        amplitude2 = norm_waveform_gyro[a]
                        break
                if latency1 > latency2:
                    g1_latency = latency2
                    g1_amplitude = amplitude2
                    loc_g1 = loc2
                    g2_latency = latency1
                    g2_amplitude = amplitude1
                    loc_g2 = loc1
                else:
                    g1_latency = latency1
                    g1_amplitude = amplitude1
                    loc_g1 = loc1
                    g2_latency = latency2
                    g2_amplitude = amplitude2
                    loc_g2 = loc2

                # Setting the sliders with angular velocity peak
positions
                slider_G1 = st.number_input(
                    "Select G1 peak", min_value=1, max_value=length-1,
value=loc_g1, step=1)
                slider_G2 = st.number_input(
                    "Select G2 peak", min_value=1, max_value=length-1,
value=loc_g2, step=1)

                # Now, we search the peak in the acceleration norm
between the task onset and 200 ms later. The value of 200 ms was
arbitrary and we observed that was suitable to the peak detection. This
peak is the acceleration peak during the sit-to-standing transition.
                standing_peak_acc = np.max(
                    norm_waveform[slider_onset:slider_onset+200])
                standing_peak_loc = 0
                for i in norm_waveform:
                    if i != standing_peak_acc:
```

```
                        standing_peak_loc = standing_peak_loc + 1
                        standing_peak_latency = t[standing_peak_loc]
                    else:
                        break

                # Now, we search the peak in the acceleration norm
between moment of the angular velocity peak of the pre-sitting turn and
the task offset. This peak is the acceleration peak during the standing-
to-sit transition.
                sitting_peak_acc =
np.max(norm_waveform[loc_g2:loc_offset])
                sitting_peak_loc = 0
                for i in norm_waveform:
                    if i != sitting_peak_acc:
                        sitting_peak_loc = sitting_peak_loc + 1
                        sitting_peak_latency = t[sitting_peak_loc]
                    else:
                        break

                # Setting the sliders with acceleration peak positions
                slider_A1 = st.number_input(
                    "Select the A1 peak", min_value=1, max_value=length-
1, value=standing_peak_loc, step=1)
                slider_A2 = st.number_input(
                    "Select the A2 peak", min_value=1, max_value=length-
1, value=sitting_peak_loc, step=1)

                # Extracting the features from iTUG
                sit_to_standing_duration = t[slider_A1] -
t_gyro[slider_onset]
                walking_to_go_duration = t_gyro[slider_G1] - t[slider_A1]
                walking_to_return_duration = t_gyro[slider_G2] - \
                    t_gyro[slider_G1]
                return_to_sit_duration = t[slider_A2] - t_gyro[slider_G2]
                standing_to_sit_duration = t_gyro[slider_offset] -
t[slider_A2]
                total_duration = sit_to_standing_duration +
walking_to_go_duration + \
                    walking_to_return_duration + return_to_sit_duration +
standing_to_sit_duration

                # Creating arrays to plot the baselines for onset and
offset detection
                shade_baseline1_x = [
                    t[slider_baseline1], t[slider_baseline1],
t[slider_baseline2], t[slider_baseline2]]
                shade_baseline1_y = [0, 0.5, 0.5, 0]
                shade_baseline2_x = [
```

```
                    t[slider_baseline3], t[slider_baseline3],
t[slider_baseline4], t[slider_baseline4]]
                shade_baseline2_y = [0, 0.5, 0.5, 0]
            with t2:
                # Plotting the gyroscope norm with iTUG stages in color
shades
                plt.figure(figsize=(5, 3))
                plt.plot(t_gyro, norm_waveform_gyro, 'k')
                plt.fill(shade_baseline1_x, shade_baseline1_y, 'b',
alpha=0.2)
                plt.fill(shade_baseline2_x, shade_baseline2_y, 'b',
alpha=0.2)
                lim_y = np.max(norm_waveform_gyro)
                shade_sitting_2_standing_x = [
                    t_gyro[slider_onset], t_gyro[slider_onset],
t[slider_A1], t[slider_A1]]
                shade_y = [0, lim_y, lim_y, 0]
                plt.fill(shade_sitting_2_standing_x, shade_y,
                         color=(1, 0.5, 0.5), alpha=0.65)
                shade_go_x = [t[slider_A1], t[slider_A1],
                              t_gyro[slider_G1], t_gyro[slider_G1]]
                plt.fill(shade_go_x, shade_y, color=(0.6, 1, 0.5),
alpha=0.65)
                shade_return_x = [
                    t_gyro[slider_G1], t_gyro[slider_G1],
t_gyro[slider_G2], t_gyro[slider_G2]]
                plt.fill(shade_return_x, shade_y,
                         color=(1, 1, 0.4), alpha=0.65)
                shade_pre_sitting_x = [
                    t_gyro[slider_G2], t_gyro[slider_G2], t[slider_A2],
t[slider_A2]]
                plt.fill(shade_pre_sitting_x, shade_y,
                         color=(0.5, 0.6, 1), alpha=0.65)
                shade_sitting_x = [t[slider_A2], t[slider_A2],
                                   t_gyro[slider_offset],
t_gyro[slider_offset]]
                plt.fill(shade_sitting_x, shade_y,
                         color=(0.4, 0.4, 0.4), alpha=0.65)
                baseline_duration = str(
                    round(t[slider_baseline2] - t[slider_baseline1], 2))
+ " s"
                plt.text(t[slider_baseline1], 0.6, baseline_duration)
                baseline_duration_offset = str(
                    round(t[slider_baseline4] - t[slider_baseline3], 2))
+ " s"
                plt.text(t[slider_baseline3], 0.6,
baseline_duration_offset)
                plt.plot([t_gyro[slider_onset], t_gyro[slider_onset]], [
                         0, lim_y], '--r')
```

```
                plt.plot([t_gyro[slider_offset], t_gyro[slider_offset]],
[
                         0, lim_y], '--b')
                plt.plot(t_gyro[slider_G1],
norm_waveform_gyro[slider_G1], marker='o',
                         markerfacecolor='none', markeredgecolor='k',
markersize=14)
                plt.text(t_gyro[slider_G1-20],
                         norm_waveform_gyro[slider_G1]*1.05, 'G1')
                plt.plot(t_gyro[slider_G2],
norm_waveform_gyro[slider_G2], marker='o',
                         markerfacecolor='none', markeredgecolor='k',
markersize=14)
                plt.text(t_gyro[slider_G2-20],
                         norm_waveform_gyro[slider_G2]*1.05, 'G2')
                plt.ylim(0, np.max(norm_waveform_gyro)*1.2)
                plt.xlabel('Time (s)')
                plt.ylabel('Angular velocity (rad/s)')
                st.pyplot(plt)

           # Plotting the accelerometer norm with iTUG stages in color
shades
                fig = plt.figure(figsize=(5, 3))
                plt.plot(t, norm_waveform, 'k')
                lim_y = np.max(norm_waveform)
                shade_y = [0, lim_y, lim_y, 0]
                plt.plot(t[slider_A1], norm_waveform[slider_A1],
marker='o',
                         markerfacecolor='none', markeredgecolor='k',
markersize=14)
                plt.text(t[slider_A1-20], norm_waveform[slider_A1]*1.05,
'A1')
                plt.plot(t[slider_A2], norm_waveform[slider_A2],
marker='o',
                         markerfacecolor='none', markeredgecolor='k',
markersize=14)
                plt.text(t[slider_A2-20], norm_waveform[slider_A2]*1.05,
'A2')
                plt.fill(shade_sitting_2_standing_x, shade_y,
                         color=(1, 0.5, 0.5), alpha=0.65)
                plt.fill(shade_go_x, shade_y, color=(0.6, 1, 0.5),
alpha=0.65)
                plt.fill(shade_return_x, shade_y,
                         color=(1, 1, 0.4), alpha=0.65)
                plt.fill(shade_pre_sitting_x, shade_y,
                         color=(0.5, 0.6, 1), alpha=0.65)
                plt.fill(shade_sitting_x, shade_y,
                         color=(0.4, 0.4, 0.4), alpha=0.65)
                plt.plot([t_gyro[slider_onset], t_gyro[slider_onset]], [
```

```
                                0, lim_y], '--r')
                    plt.plot([t_gyro[slider_offset], t_gyro[slider_offset]],
[
                                0, lim_y], '--b')
                    plt.xlabel('Time (s)')
                    plt.ylabel('Acceleration (g)')
                    st.pyplot(plt)
               # Priting the feature values
                with t3:
                    st.markdown("**Outcomes**")
                    st.text('Total duration (s) = ' +
                            str(round(total_duration, 2)))
                    st.text('Sit to stand duration (s) = ' +
                            str(round(sit_to_standing_duration, 2)))
                    st.text('Go walk duration (s) = ' +
                            str(round(walking_to_go_duration, 2)))
                    st.text('Return walk duration (s) = ' +
                            str(round(walking_to_return_duration, 2)))
                    st.text('Stand to sit duration (s) = ' +
                            str(round(standing_to_sit_duration, 2)))
                    st.text('Standing acceleration peak (g) = ' +
                            str(round(norm_waveform[slider_A1], 2)))
                    st.text('Sitting acceleration peak (g) = ' +
                            str(round(norm_waveform[slider_A2], 2)))
                    st.text('Return turning amplitude (rad/s) = ' +
                            str(round(norm_waveform_gyro[slider_G1], 2)))
                    st.text('Pre-sit turning amplitude (rad/s) = ' +
                            str(round(norm_waveform_gyro[slider_G2], 2)))
```

REFERENCE

Ko, J. B., Hong, J. S., Shin, Y. S., & Kim, K. B. (2022). Machine Learning-Based Predicted Age of the Elderly on the Instrumented Timed Up and Go Test and Six-Minute Walk Test. Sensors (Basel, Switzerland), 22(16), 5957. https://doi.org/10.3390/s22165957

Van Lummel, R. C., Ainsworth, E., Lindemann, U., Zijlstra, W., Chiari, L., Van Campen, P., & Hausdorff, J. M. (2013). Automated approach for quantifying the repeated sit-to-stand using one body fixed sensor in young and older adults. Gait & posture, 38(1), 153–156. https://doi.org/10.1016/j.gaitpost.2012.10.008
